# Preoperative Risk Factors for Pain After Reverse Total Shoulder Arthroplasty: A Systematic Review

**DOI:** 10.7759/cureus.60041

**Published:** 2024-05-10

**Authors:** Isa Waheed, Fenu Ediripolage, Isaamuddin Alvi, Jawwad Mihran Haider

**Affiliations:** 1 Department of Trauma and Orthopaedics, Imperial College Healthcare NHS Trust, London, GBR; 2 Department of Urology, St George's University Hospitals NHS Foundation Trust, London, GBR; 3 Department of General Surgery, Oxford University Hospitals NHS Foundation Trust, Oxford, GBR; 4 Department of General Surgery, Chelsea and Westminster Hospital NHS Trust, London, GBR

**Keywords:** preoperative care, preop planning, : pain, predictive factor, reverse shoulder arthoplasty

## Abstract

Despite being a generally successful procedure, pain following reverse total shoulder arthroplasty (rTSA) is a known complication. The aim of this systematic review is to identify preoperative risk factors for pain following rTSA to encourage evidence-based interventions, inform clinicians, and aid in surgical planning. Studies that reported preoperative risk factors and pain after rTSA were included. Studies which reported outcome measures that incorporated pain scores yet did not display them independently, studies which only reported intraoperative risk factors, and studies involving participants under 18 were excluded. The search was conducted on May 31, 2023, across the following databases: PubMed, Web of Science, Embase, Scopus, and Cochrane Central Register of Controlled Trials. Four independent researchers conducted this systematic review, and a descriptive analysis was subsequently performed. Twenty-five studies were included following the evaluation of full-text articles, involving a total of 9,470 shoulders. Preoperative risk factors identified were categorised into the following groups: BMI, smoking, radiographic findings, age and sex, prior surgery, functional ability and pain, and psychosocial. The strongest associations identified were preoperative opioid use and smoking, which were both associated with worse pain outcomes following rTSA; other preoperative risk factors highlighted in this review showed either weak or no correlation. Preoperative opioid use and smoking are likely risk factors for the development of pain after rTSA. Although the studies included varying levels of quality, the identification of modifiable risk factors is useful in optimising management prior to surgery and guiding patient expectations. The lack of evidence regarding associations with non-modifiable risk factors further reinforces the potential benefits of the procedure on diverse population groups and is useful in itself for assessing the candidacy of patients for the procedure, particularly when postoperative pain is a factor being considered.

## Introduction and background

Shoulder arthroplasty has evolved into a highly efficacious surgical intervention for addressing various debilitating conditions affecting the glenohumeral joint, such as osteoarthritis and rotator cuff tears [1]. Despite advancements in surgical techniques and implant designs, the effective management of pain following shoulder arthroplasty remains imperative for optimal patient outcomes. Uncontrolled or persistent pain can impede rehabilitation, compromise functional recovery, and influence overall patient satisfaction [2].

As the demand for shoulder arthroplasty continues to rise, it becomes essential to comprehend the multifactorial nature of postoperative pain. Numerous variables contribute to the variability in pain experiences among patients undergoing shoulder arthroplasty, encompassing preoperative conditions, surgical factors, and postoperative care strategies [3].

Reverse total shoulder arthroplasty (rTSA) is a specific type of shoulder joint replacement whereby a metal ball is fixed to the shoulder socket and a plastic cup to the humerus, contrary to the natural anatomy of the shoulder joint and conventional TSA. It has emerged as an effective method for managing pain and optimising shoulder function for a range of pathologies; between 2005 and 2015, the proportion of shoulder arthroplasty surgeries performed in the USA classified as rTSAs grew from 27% to 52%, and this proportion is projected to increase [4]. One in five patients develops persistent pain following TSA; although rTSA generally yields better outcomes, persistent pain is still a documented phenomenon [5].

This systematic review seeks to comprehensively analyze the existing literature on preoperative risk factors associated with pain after rTSA. By synthesising the available evidence, our objective is to elucidate the intricate interplay of patient-related factors that contribute to the development and persistence of pain in the post-arthroplasty period. Through a thorough analysis of the current literature, we aim to provide clinicians, researchers, and healthcare stakeholders with a nuanced understanding of the key preoperative determinants of postoperative pain, ultimately informing evidence-based interventions and improving the overall quality of care for individuals undergoing rTSA. 

To our knowledge, this is the first systematic review exploring preoperative risk factors of pain following rTSA. The exploration of both modifiable and non-modifiable risk factors for post-rTSA pain not only addresses a critical gap in the existing literature but also lays the groundwork for developing targeted interventions to minimise pain, enhance patient satisfaction, and optimise the long-term success of shoulder arthroplasty procedures [6]. As the field continues to evolve, the insights derived from this systematic review will contribute to a more comprehensive and evidence-driven approach to managing pain in the context of rTSA, fostering advancements in patient care and outcomes. 

## Review

Methods

This systematic review was conducted following the Preferred Reporting Items for Systematic Reviews and Meta-Analyses (PRISMA) guidelines [7].

Search Strategy

The search was conducted on 31 May 2023 across the following databases: PubMed, Web of Science, Embase, Scopus, and Cochrane Central Register of Controlled Trials. The search terms were adapted for each database to narrow down the area of focus, and only full-text results were included. The search terms included: (Shoulder) AND (Arthroplast* OR "replacement"[Mesh] OR "arthroplasty, replacement, shoulder"[Mesh]) AND (after OR continue OR post OR recur OR ongoing OR chronic OR persistent OR long term) AND (risk OR predict OR factor OR associat* OR correlat* OR effect OR affect OR influence). There were no restrictions placed on the year of publication, and all languages were included.

Eligibility Criteria

Inclusion criteria were as follows: (1) evaluating preoperative risk factors in comparison to a control group, (2) reporting pain scores or opioid use after rTSA, (3) any indications for rTSA, (4) having a follow-up period of at least 12 weeks, (5) being published at any time up to the search date, and (6) including participants of any sex. 

Exclusion criteria were as follows: (1) studies which reported outcome measures that incorporated pain scores yet did not display them independently, (2) studies which only reported intraoperative risk factors, and (3) studies involving participants under the age of 18. 

This literature review was conducted by four independent researchers. Abstract screening and full-text review were conducted using the online platform Covidence, and a PRISMA flow diagram was generated [8].

Quality Assessment

Bias assessment criteria from a previous systematic review assessing risk factors for pain after hip arthroplasty were used [9], as these were found to be suitably tailored to the studies reviewed and more objective in categorising risk. 

The criteria applied were (1) adequate adjustment to minimise confounding through the use of multivariate or univariate analysis; (2) selection of patients from multiple centres rather than a single institution; (3) patients were selected consecutively; and (4) <20% of patients lost to follow-up if follow-up was less than one year or <30% if follow-up was greater than one year. Studies were considered low risk if they met three or four criteria; studies that met two criteria were considered medium risk; and studies that met one or no criteria were considered high risk. Two reviewers independently evaluated the risk of bias in the included studies, with any discrepancies being resolved after a consensus meeting. Any studies in which accordance with the above criteria was unclear were assumed to have not met them.

Data Extraction and Analysis

Abstract screening and full-text review were conducted using the online platform Covidence, and a PRISMA flow diagram was generated. A carefully performed two-stage process was used for data extraction to ensure a high degree of accuracy. The first stage involved screening titles and abstracts to assess their relevance to the basis of the review; this was carried out by four independent researchers for each title and abstract screened. Articles that were considered eligible for the second stage were assessed as having met an acceptable degree of relevance. The second stage involved assessing full-text articles, each assessed by four independent researchers, and those that met the inclusion criteria were selected for a comprehensive process of data extraction. A uniform framework for data extraction, made using Microsoft Excel, was used to collect and categorise important information from each paper. Consensus decision-making was used to resolve discrepancies between reviewers following this process.

Data extracted included title, author(s), publication year, study design, sample size, preoperative risk factors, follow-up period, postoperative pain outcomes, associations, and P values for the level of significance. Preoperative risk factors were categorised into the following groups: BMI, smoking, radiographic findings, age and sex, prior surgery, functional ability and pain, and psychosocial. Unfortunately, the high heterogeneity of outcome measures and study designs prevented us from conducting a meaningful meta-analysis. Therefore, the chosen approach was a narrative synthesis.

Results

**Figure 1:**
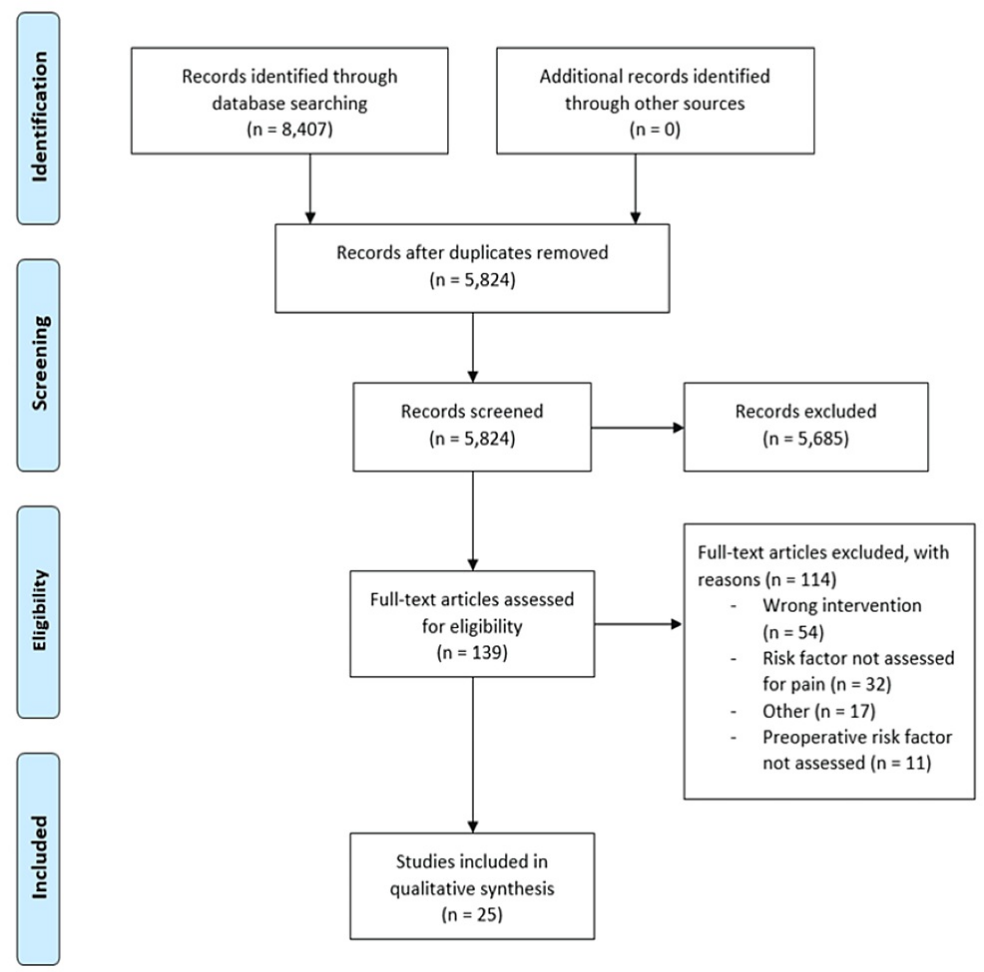
Flow diagram of a systematic review. Databases searched include PubMed, Web of Science, Embase, Scopus, and Cochrane Central Register of Controlled Trials.

A total of 8,407, including duplicates, were identified from online databases and registers. Of these, the full text review was carried out on 139 publications. Using our inclusion criteria, this left 25 remaining studies (Figure 1). All the studies included were observational studies and used retrospective cohort data. The sample sizes varied between 35 and 2,133 shoulders, with a total of 9,470 shoulders assessed. All rTSAs were performed in 2005 or later. The risk factors that were commonly evaluated included BMI (two studies), smoking (two studies), radiographic changes (nine studies), age and gender (four studies), prior surgery (three studies), functional ability and pain (five studies), and psychosocial (three studies). Outcomes were assessed using the visual analogue scale (VAS), American Shoulder and Elbow Surgeons (ASES) score, Shoulder Pain and Disability Index (SPADI), Constant-Murley score, and postoperative opioid usage.

Following assessment of bias, eight studies were considered to have a high risk of bias, seven were considered to have a medium risk, and 10 were considered to have a low risk (Table 1). Assessing whether patient selection was consecutive, or if follow-up was adequate, was not possible for several studies due to their retrospective nature. 

**Table 1:** Risk of bias among 25 studies of adult rTSA. rTSA: reverse total shoulder arthroplasty. *"-" *denotes unable to assess*.*

First author	Year	Consecutive patient selection	Multicentre enrolment	Adequate follow-up	Adjustment for confounders	Risk of bias
Andryk [10]	2023	Yes	No	Yes	Yes	Low
Baessler [11]	2022	-	No	-	Yes	High
Beck [12]	2013	Yes	No	-	Yes	Medium
Berthold [13]	2021	-	Yes	No	No	High
Blaber [14]	2022	Yes	No	Yes	Yes	Low
Boettcher [15]	2022	-	Yes	-	No	High
Burrus [16]	2022	-	Yes	-	Yes	Medium
Dean [17]	2022	-	No	Yes	Yes	Medium
Hartline [18]	2021	-	No	No	Yes	High
Hung [19]	2021	Yes	No	No	Yes	Medium
Jang [20]	2019	Yes	No	No	No	High
Kääb [21]	2022	Yes	Yes	Yes	No	Low
Lansdown [22]	2020	-	No	No	Yes	High
Marigi [23]	2022	Yes	Yes	-	Yes	Low
Mcfarland [24]	2021	Yes	No	Yes	Yes	Low
Moore [25]	2021	Yes	No	Yes	Yes	Low
Morris [26]	2015	Yes	No	-	Yes	Medium
Neel [27]	2022	Yes	Yes	Yes	Yes	Low
Parsons [28]	2020	Yes	Yes	-	Yes	Low
Puzzitiello [29]	2021	-	No	Yes	No	High
Rauck [30]	2018	Yes	No	No	Yes	Medium
Saini [31]	2022	Yes	No	Yes	Yes	Low
Walters [32]	2020	Yes	No	Yes	Yes	Low
Wong [33]	2017	-	No	-	Yes	High
Yoon [34]	2017	-	No	Yes	Yes	Medium

Body Mass Index

We found two studies that looked at the association between BMI and postoperative pain following rTSA (Table 2), with one paper comparing patients in the normal weight, overweight, and obese categories specifically. Both studies concluded that there was no clinically significant correlation between BMI and pain following rTSA.

**Table 2:** Associations of BMI with pain following rTSA. ASES: American Shoulder and Elbow Surgeons; n: number of shoulder; RC: retrospective cohort; VAS: Visual Analogue Scale; rTSA: reverse total shoulder arthroplasty.

First author	Year	Study design	n	Follow-up	Outcome measures	Associations
Beck [12]	2013	RC	76	Two years	VAS	No statistically significant association between BMI (ranging from 18.5 to >30) and pain following rTSA
Hung [19]	2021	RC	88	Two years	ASES pain subscore	No statistically significant association between BMI and pain following rTSA

Smoking

We found two studies that looked at the association between smoking and postoperative pain following rTSA (Table 3). Hartline et al. [18] investigated tobacco usage as a risk factor for postoperative pain in 279 cases. They found that tobacco users were found to report significantly more pain at the postoperative 12-week mark when compared to non-users. No significant difference was found in the improvement of pain scores from pre- to post-op between the two groups. Tobacco users also showed a greater increase in postoperative opioid use, with a 2,643 mg oral morphine equivalents (OME) increase compared to a 2,121 mg OME increase in non-users.

**Table 3:** Associations of tobacco use with pain following rTSA. RC: retrospective cohort; n: number of shoulders; VAS: Visual Analogue Scale; rTSA: reverse total shoulder arthroplasty.

First author	Study design	n	Follow-up	Outcome measures	Associations
Hartline [18]	RC	279	12 weeks	VAS for pain, opioid usage	Tobacco users reported significantly more pain and greater opioid use postoperatively than non-users
Walters [32]	RC	186	Two years	VAS for pain	Smokers reported significantly more pain postoperatively than both non-users and former users

An earlier retrospective study in 2020 by Walters et al. [32] reported similar results. Of note, there was a significant difference in age between the smoker (mean age 62.1 years) and non-smoker groups (mean age 70.7 years). Smokers were found to have significantly higher VAS pain scores after a two-year minimum follow-up (range 2-5.7 years) than both non-smokers and former smokers.

Radiographic Findings

We found nine studies that looked at the association between radiographic findings and postoperative pain following rTSA (Table 4). Berthold et al. used AP radiographs to measure centre of rotation (COR), critical shoulder angle (CSA), acromiohumeral distance (AHD), lateral humeral offset (LHO), glenoid inclination (GI), and lateralisation shoulder angle (LSA), revealing a significant association between lower preoperative LSA and higher postoperative pain [13]. Meanwhile, Saini et al. demonstrated that a preoperative diagnosis of rotator cuff tear arthropathy (CTA) is associated with greater postoperative pain when compared to rTSA with a GHOA diagnosis [31]. Furthermore, Yoon et al. found that preoperative deltoid muscle volume positively correlates with postoperative pain, and this was statistically significant [34].

**Table 4:** Associations of radiographic findings with pain following rTSA. ASES: American Shoulder and Elbow Surgeons; CTA: cuff tear arthropathy; GC: Goutallier classification; GHOA: glenohumeral osteoarthritis; n: number of shoulders; RC: retrospective cohort; LSA: lateralisation shoulder angle; VAS: visual analogue scale; rTSA: reverse total shoulder arthroplasty.

First author	Year	Study design	n	Follow-up	Outcome measures	Associations
Berthold [13]	2021	RC	61	Two years	VAS	Statistically significant association between preoperative LSA and postoperative pain
Hung [19]	2021	RC	88	Two years	ASES pain subscore	No statistically significant association between muscle fatty infiltration (GC classification) and postoperative ASES pain subscore. No statistically significant association between rotator cuff tear size and the ASES pain subscore
Jang [20]	2019	RC	90	One year	VAS	No statistically significant association between teres minor hypertrophy and postoperative VAS
Kääb [21]	2022	Multicentre Observation study	202	Two years	VAS	No statistically significant association between radiographic Hamada classification and postoperative VAS score
Lansdown [22]	2020	RC	177	Two years	ASES pain subscore	No statistically significant association between preoperative glenoid retroversion and postoperative ASES pain subscore
Puzzitiello [29]	2021	RC	81	Two years	VAS	No statistically significant association between rotator cuff fatty infiltration and postoperative VAS score
Saini [31]	2022	RC	311	Two years	VAS	Statistically significant correlation between CTA diagnosis and greater postoperative pain when compared to GHOA diagnosis
Dean [17]	2022	RC	262	Three years	VAS	No statistically significant correlation between radiographic Hamada findings and postoperative VAS score
Yoon [34]	2017	RC	35	One year	VAS	Statistically significant positive association between poor deltoid muscle mass and postoperative VAS score

The remaining six studies found no significant association with postoperative pain. These preoperative factors that were assessed included: muscle fatty infiltration into the periarticular musculature of the shoulder [19,29], teres minor hypertrophy [20], glenoid retroversion [22] and the Hamada classification system [17,21].

Age and Sex

We found three studies that looked at the association between patient age and postoperative pain following rTSA (Table 5). Of these studies, one found no association between patient age and postoperative pain following rTSA [15], one found a positive relationship between age and postoperative pain [19], and the third found that patients younger than 60 reported greater levels of pain on a daily basis postoperatively when compared to patients 60-79 years old [27]. Only two studies looked at the association between patient gender and postoperative pain following rTSA; no statistically significant difference was found in either [19,33].

**Table 5:** Associations of age and sex with pain following rTSA. ASES: American Shoulder and Elbow Surgeons; n: number of shoulders; RC: retrospective cohort; VAS: visual analogue scale; rTSA: reverse total shoulder arthroplasty.

First author	Year	Study design	n	Follow-up	Outcome measures	Associations
Boettcher [15]	2022	RC	2,133	Two years	VAS for pain	No statistically significant difference in daily basis pain postoperatively between patients aged 60-79 and patients aged ≥80
Hung [19]	2021	RC	88	Two years	ASES pain subscore	Statistically significant positive relationship between age and postoperative pain. No statistically significant difference in postoperative pain between male and female patients
Neel [27]	2022	RC	1,917	Two years	VAS for pain	Patients aged <60 years report significantly more pain on a daily basis postoperatively compared to patients in the 60-79 years age group
Wong [33]	2017	RC	117	One year	ASES pain and VAS for pain	No statistically significant difference in postoperative pain between male and female patients

Prior Surgery

We found three studies that looked at the association between prior surgery and postoperative pain following rTSA (Table 6), of which two looked at prior rotator cuff repair (RCR). One study found that prior RCR was associated with greater postoperative pain and less improvement in pain following rTSA [23], while another study found no association between prior RCR and postoperative pain following rTSA [17]. The third study found no association between prior arthroscopic acromioplasty and postoperative pain following rTSA [14].

**Table 6:** Associations of prior surgery with pain following rTSA. CC: case-control; n: number of shoulders; ORIF: open reduction internal fixation; RC: retrospective cohort; RCR: rotator cuff repair; SPADI: Shoulder Pain and Disability Index; VAS: visual analogue scale; rTSA: reverse total shoulder arthroplasty.

First author	Year	Study design	n	Follow-up	Outcome measures	Associations
Blaber [14]	2022	RC	90	Two years	VAS for pain	No statistically significant association between prior arthroscopic acromioplasty and pain following rTSA
Dean [17]	2021	RC	192	Two years	VAS for pain	No statistically significant association between prior RCR and pain following rTSA
Marigi [23]	2022	RC	1,314	Two years	VAS for pain	Prior RCR is associated with greater postoperative pain and less improvement in pain following rTSA

Functional Ability and Pain

We found five studies assessing preoperative pain or function and its impact on postoperative pain (Table 7). One concluded that there was no statistically significant difference in postoperative pain following rTSA between upper extremity ambulators and controls [10]. Another study found no statistically significant difference in postoperative pain between patients with preserved and restricted forward elevation preoperatively [16]. One study looking at preoperative external rotation deficit also found no statistically significant difference in postoperative pain [28].

**Table 7:** Associations of pain and functional ability with pain following rTSA. aER: active external rotation; ASES: American Shoulder and Elbow Surgeons; FE: forward elevation; n: number of shoulders; RC: retrospective cohort; VAS: visual analogue scale; rTSA: reverse total shoulder arthroplasty.

First author	Year	Study design	n	Follow-up	Outcome measure	Associations
Andryk [10]	2023	RC	159	Two years	VAS for pain	No statistically significant difference in postoperative pain between upper extremity ambulator patients and control
Baessler [11]	2022	RC	264	Two years	VAS for pain	Preoperative opioid use associated with higher VAS scores when compared to opioid-naive patients
Burrus [16]	2022	RC	81	Two years	VAS for pain	No statistical significant difference in postoperative pain between patients who underwent rTSA with preserved preoperative FE and restricted preoperative FE
Morris [26]	2015	RC	68	Two years	Constant pain score and ASES pain subscore	Preoperative opioid use is associated with worse Constant pain score postoperatively and worse ASES-pain subscore postoperatively
Parsons [28]	2020	RC	1,154	Two years	VAS for pain	No statistically significant difference in postoperative pain between patients with aER deficits and patients with no aER deficit

We found two studies assessing preoperative pain in the form of opioid use [11,26]. They concluded that patients with preoperative opioid use had inferior VAS pain scores postoperatively compared to opioid-naive patients (2.59 vs. 1.25, respectively). In addition, they had worse Constant pain and ASES pain sub-scores.

Psychosocial

We found three studies looking at the relationship between various preoperative psychosocial factors and postoperative pain (Table 8). One study compared patients receiving Social Security Disability Insurance or worker’s compensation with a control group [24]. This concluded that there was indeed a statistically significant positive relationship between the aforementioned factors and postoperative pain following rTSA. A separate study exploring preoperative patient expectations found that patients with higher expectations preoperatively had better postoperative pain relief at night [30]. Moore et al., who assessed the impact of mental health disorders on postoperative outcomes, found no statistically significant difference in postoperative pain between patients with anxiety or depression and patients without [25].

**Table 8:** Associations of psychosocial factors with pain following rTSA. n: number of shoulders; RC: retrospective cohort; VAS: visual analogue scale; rTSA: reverse total shoulder arthroplasty.

First author	Year	Study design	n	Follow up	Outcome measure	Association
McFarland [24]	2021	RC	125	Two years	VAS for pain	Statistically significant positive relationship between Social Security Disability Insurance/workers’ compensation and postoperative pain
Moore [25]	2021	RC	114	Two years	VAS for pain	No statistically significant difference in postoperative pain between patient group with anxiety/depressive disorder and control
Rauck [30]	2018	RC	135	Two years	VAS for pain	Statistically significant negative relationship between preoperative expectations and postoperative pain at night only

Discussion

The most consistent preoperative risk factors associated with poor postoperative pain outcomes were tobacco and opioid use. At all follow-ups across both included studies, VAS pain scores were consistently worse among tobacco users when compared to controls. In addition, it was found that tobacco users had higher postoperative analgesic requirements compared to control groups. One possible explanation for this is that prolonged smoking can lead to desensitisation of nicotinic acetylcholine receptors (nAChRs), which have a role in minimising chronic pain by regulating neuroinflammation [35,36]. A consistent association was also found regarding preoperative opioid usage, with all studies showing opioid use leading to inferior VAS pain score, Constant pain score, and ASES pain sub-scores compared to opioid-naive groups following rTSA. Opioid-induced hyperalgesia is a known phenomenon that has been documented in the literature. A previous study by Chen et al. used quantitative sensory testing to demonstrate that opioid use leads to a paradoxical decrease in the nociceptive threshold [37]. However, whether this is the mechanism leading to greater pain among opioid users specifically in the postoperative period cannot be stated for certain, as research into the subject is scarce. 

Although these data should have no influence on patient selection prior to rTSA, they highlight two groups that may benefit from preoperative optimisation. As the studies demonstrated better postoperative pain among former smokers compared to current smokers, this suggests smoking cessation or reduction would be of benefit by allowing for better short-term pain management and improved postoperative outcomes [38]. A recent systematic review by Harrogate et al. assessing perioperative smoking cessation interventions highlights the effectiveness these programs have at facilitating smoking cessation, both at the time of surgery and 12 months after surgery [39]. In addition, patients with chronic pain and long-term opioid use may benefit from pain clinic referrals prior to surgical intervention to optimise their analgesic use and consider attempting opioid weaning [40]. 

It should be noted that in both studies assessing the effect of smoking, there was an uneven age distribution among groups, with smokers making up a considerably smaller proportion of total patients included compared to former smokers and non-smokers. However, we believe this age discrepancy is not of major significance as poor correlations were found between age and postoperative pain, with all three studies assessing age as a preoperative risk factor showing different associations with postoperative pain. Previous studies seem to corroborate this unclear relationship, with inconsistent associations between age and postoperative pain existing across a broad range of surgical procedures [41,42]. Although older patients may have greater comorbidities and reduced recovery [43], younger patients tend to have greater postoperative activity levels, which may contribute to increased pain. With conflicting evidence, further research would be beneficial. 

No associations were found between most radiographic findings assessed and postoperative pain following rTSA, with only three out of nine studies highlighting statistically significant correlations. An interesting finding from one study was that greater postoperative pain was associated with lower deltoid muscle mass; a previous study by Tungtrongjik et al. investigating the effect preoperative quadriceps exercise on outcomes following total knee arthroplasty showed that patients in the exercise group experienced reduced postoperative pain when compared to controls [44]. Although the same cannot be definitively concluded for deltoid exercises based on the current data, this highlights an interesting avenue for further research. LSA and CTA diagnosis were also associated with higher postoperative pain, although without additional studies echoing these conclusions, the results may be weak. Associations between previous shoulder surgery and pain following rTSA were also weak, with studies showing mixed evidence regarding the impact of prior RCR repair. This unclear relationship is made further difficult to assess by the heterogeneity of surgical techniques and intraoperative variables of prior surgeries; no reliable associations could feasibly be made based on the data available.

When assessing the impact of psychosocial factors on pain following rTSA, our data revealed no difference in pain between control groups and patients with psychiatric conditions. The data did, however, highlight that having higher expectations preoperatively led to reduced nighttime postoperative pain. Reduction in nighttime pain following shoulder surgery has important implications due to its association with improving sleep quality [45], further reinforcing the importance of preoperative educational sessions, which have already demonstrated the ability to broadly improve outcomes and satisfaction [46], to address patients’ concerns and optimise expectations. Although a correlation was established between worse pain scores following rTSA and receiving disability insurance or worker's compensation, only one study was assessed, highlighting another potential area for further research. This could be largely due to reduced resources and likely discrepancies in access to health care, unfortunately leading to delayed presentation, advanced disease, and worse surgical outcomes [47]. 

No association was established between preoperative shoulder function and postoperative pain in all studies included. Similar results were found for preoperative BMI, with no statistically significant difference in pain following rTSA across varying BMIs as well as patient sex. The lack of association with BMI seems to reflect previous studies, which have shown improvements in pain are similar among patients following arthroplasty procedures across a variety of joints regardless of BMI, including total knee arthroplasty [48] as well as hip arthroplasty [9]. These preoperative factors showing little association with postoperative pain provide further evidence for rTSA being an efficacious intervention for a wide spectrum of patient demographics.

This systematic review is not without its limitations. Notably, eight out of the 25 studies exhibited a high risk of bias. Although this was due to a scarcity of papers available for inclusion, this bias can cause our estimates of association to be either larger or smaller than the true association. Additionally, the included studies were observational and retrospective in nature, encumbering the ability to assess patient selection, loss of follow-up, and confidently demonstrate causality. Most studies employed multivariate analysis to adjust for confounders during data analysis, but five did not, impeding reliability and validity. Moreover, the maximum follow-up period was two to three years, so our data cannot be used to predict pain outcomes beyond this timeframe. High heterogeneity among studies in terms of outcome measures made performing a meta-analysis infeasible, diminishing the accuracy of results.

Despite all studies assessing patients' pain before and after surgery, they do not specify whether the location and type of postoperative pain are the same as preoperatively in their assessments. The question remains as to whether patients are reporting new pain or persistence of the same pain. This provides an avenue for further research. Other limitations include detection bias due to differences in postoperative assessment methods used and reporting bias with the use of subjective patient-reported outcome measures. This review underscores the need for further research with low-risk-of-bias standardised designs, and objective core outcome sets (COS) that are uniform to enable high-quality meta-analysis and provide stronger conclusions on the preoperative risk factors for pain after rTSA.

## Conclusions

Although the establishment of strong conclusions is made difficult by the limited number of studies available for each preoperative risk factor assessed, as well as the variance in quality, a number of factors have been identified that show some evidence of being associated with greater postoperative pain following rTSA. Modifiable factors identified, such as smoking and opioid use, provide scope for lifestyle changes to be made preoperatively to mitigate postoperative pain, while non-modifiable factors showing no relationship may aid in consideration of management for a wider set of demographics.
